# Id1 Restrains p21 Expression to Control Endothelial Progenitor Cell Formation

**DOI:** 10.1371/journal.pone.0001338

**Published:** 2007-12-19

**Authors:** Alessia Ciarrocchi, Vladimir Jankovic, Yuval Shaked, Daniel J. Nolan, Vivek Mittal, Robert S. Kerbel, Stephen D. Nimer, Robert Benezra

**Affiliations:** 1 Program of Cancer Biology and Genetics, Memorial Sloan-Kettering Cancer Center, New York, New York, United States of America; 2 Program of Molecular Pharmacology and Chemistry, Memorial Sloan-Kettering Cancer Center, New York, New York, United States of America; 3 Molecular and Cellular Biology Research, Sunnybrook Health Sciences Centre, Toronto, Ontario, Canada; 4 Cancer Genome Research Center, Cold Spring Harbor Laboratory, Woodbury, New York, United States of America; Katholieke Universiteit Leuven, Belgium

## Abstract

Loss of Id1 in the bone marrow (BM) severely impairs tumor angiogenesis resulting in significant inhibition of tumor growth. This phenotype has been associated with the absence of circulating endothelial progenitor cells (EPCs) in the peripheral blood of Id1 mutant mice. However, the manner in which Id1 loss in the BM controls EPC generation or mobilization is largely unknown. Using genetically modified mouse models we demonstrate here that the generation of EPCs in the BM depends on the ability of Id1 to restrain the expression of its target gene p21. Through a series of cellular and functional studies we show that the increased myeloid commitment of BM stem cells and the absence of EPCs in Id1 knockout mice are associated with elevated p21 expression. Genetic ablation of p21 rescues the EPC population in the Id1 null animals, re-establishing functional BM-derived angiogenesis and restoring normal tumor growth. These results demonstrate that the restraint of p21 expression by Id1 is one key element of its activity in facilitating the generation of EPCs in the BM and highlight the critical role these cells play in tumor angiogenesis.

## Introduction

Bone marrow-derived hematopoietic and endothelial cells contribute to tumor angiogenesis. Whereas hematopoietic cells promote tumor angiogenesis in a paracrine manner by releasing pro-angiogenic factors and creating permissive conditions in the tumor microenvironment [1]–[4], EPCs are incorporated into nascent blood vessels and differentiate into mature endothelial cells [5]–[7]. In the past decade a number of reports have described the incorporation of BM-derived EPCs into tumor vessels in both spontaneous murine tumor models and human patients [6], [7] but the extent of incorporation and the functional importance of these cells is still under intense debate. Our recent work has reconciled some of these issues by showing that these cells are recruited to the tumor site at very early stages of angiogenesis and are eventually diluted or replaced by mature endothelial cells from the neighboring vasculature. Ablation of these cells by radiolabeled antibodies against an EPC-specific vascular endothelial-cadherin (VE-Cadherin) epitope results in abnormal vasculature formation and delayed tumor growth [8], [9]. In addition, we recently demonstrated that BM-derived EPCs play a critical role in promoting vascular remodeling and tumor re-growth after treatment with vascular disrupting agents [10]. This further confirms the critical role of these cells at the very early stages of tumor angiogenesis and underlines the importance of developing effective strategies to inhibit the recruitment of these cells to the tumor site and block their pro-angiogenic function. However, the mechanisms that govern the behavior of these cells, from their origin in the BM to their release into the circulation in response to pro-angiogenic stimuli are still poorly understood.

Id1 is a member of a family of 4 proteins (Id1-4) known to inhibit the activity of basic helix loop helix transcription factors by restraining their ability to bind DNA [11], [12]. Loss of Id1 in the BM leads to a complete loss of the EPC population in the peripheral blood, which has been correlated with a block in tumor neovascularization and delayed tumor growth [5], [10]. However, the actual role of Id1 in regulating EPC formation or mobilization remains unknown. We recently reported that the absence of Id1 compromises the self-renewing capacity of hematopoietic stem cells (HSCs) in the BM, increasing their tendency to differentiate towards the myeloid lineage. This functional defect is associated with transcriptional changes in Id1 null HSCs, including the increased expression of p21, a well-established target of Id1 repression [13]. Here we show that Id1 is required for the previously described ability of phenotypic HSCs (Lineage- (Lin-) cKit+Sca-1+ cells) [14] to give rise to endothelial progeny [15]–[17] and demonstrate the opposing effects of Id1 and its target gene p21 on endothelial and myeloid lineage differentiation in the HSC subset. Ablation of p21 in the Id1-/- animals restores a functional endothelial population, rescues the angiogenic defect observed in the Id1-/- mice, and reverses the premature myeloid commitment of Id1 null HSCs. These data describe for the first time a genetic interaction between Id1 and p21 in regulating the genesis of EPCs in the BM, thereby advancing our understanding of the biology of these cells.

## Results

### Id1 expression is upregulated in BM progenitor cells, during angiogenesis

**Figure 1:**
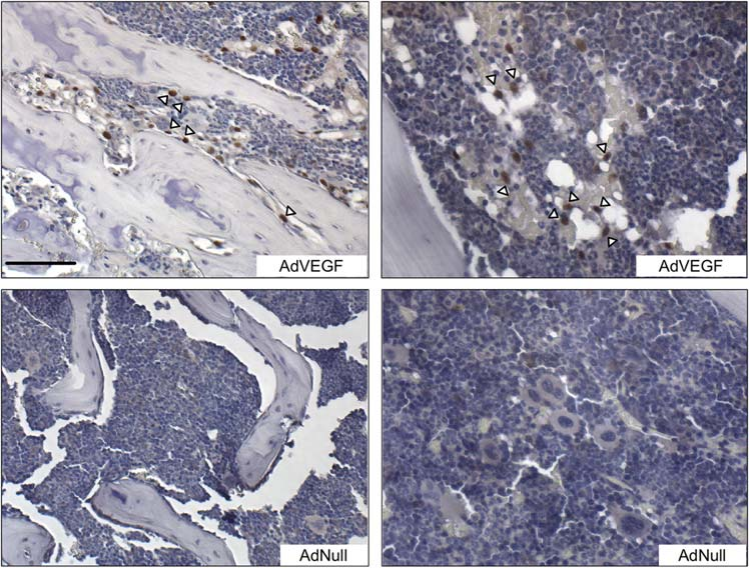
Angiogensis induces upregulation of Id1 in BM progenitor cells. A) Immunohistochemical analysis of Id1 protein in femur sections from mice treated with AdVEGF165 or with AdNull. Arrowheads indicate Id1-expressing cells in endosteal (left) and vascular (right) stem cell niche. Scale bar 100 µm.

Id1 expression is confined to the primitive progenitor cells (lineage marker negative, or Lin- subset) in resting BM [13]. To identify Id1-expressing cells in the BM upon a vascular endothelial growth factor (VEGF) challenge, we injected mice with an adenovirus encoding VEGF165 and an empty adenoviral control (AdNull)(Figure 1). AdVEGF165 administration induces a dramatic upregulation of Id1 in a subset of cells comprising less than 0.5% of the total BM population. The Id1 positive cells were either associated with the endosteal surface surrounding the BM (Figure 1a left) or with the sinusoidal endothelium in the vascular zone (Figure 1a right) both previously described as potential stem cell niches [18]–[20]. The same pattern of Id1 expressing BM cells was observed 4 days after tumor implantation (data not shown). Quantitative real time PCR and western blot analysis confirmed that Id1 is upregulated in BM progenitor cells in response to tumor growth (Figures S1a and S1b) further demonstrating that angiogenesis stimulates Id1 expression in a small subset of BM progenitor cells.

### Loss of Id1 impairs generation of EPCs in the BM

**Figure 2:**
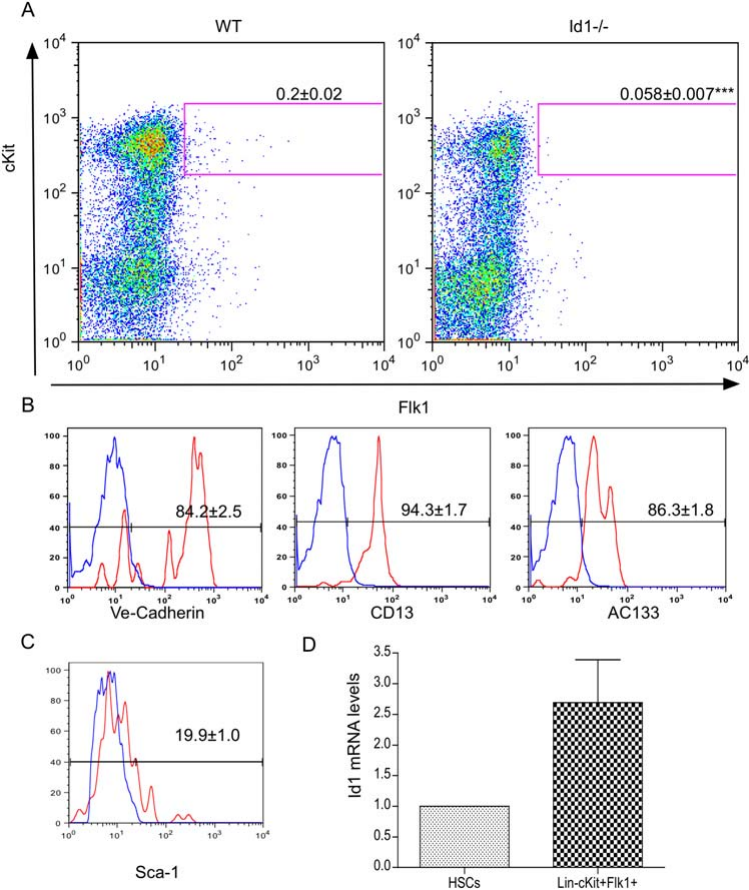
Loss of Id1 impairs generation of EPCs in the BM. A) Flow cytometric analysis of the EPC fraction of BM cells from wild type (WT) or Id1-/- mice. The profiles shown are representative of Lin- cells. The gates identified the Lin- c-kit+ Flk-1+ EPC population. The numbers indicate the average percentage±SEM of c-kit+ Flk-1+ in the Lin- fraction (n = 24, ***p<0.001). B–C) Flow cytometric analysis of the endothelial specific markers VE-cadherin, CD13, AC133 (B) and the stem cell specific marker Sca-1(C) in the Lin- c-kit+ Flk-1+ population. The histograms represent the distribution of Lin- c-kit+ Flk-1+ stained with the specific marker indicated (red) or the isotype control (blue). The numbers indicate the average percentage±SEM of marker positive cells in the Lin- c-kit+ Flk-1+ fraction (n = 5). D) Quantitative real time PCR analysis of Id1 mRNA levels in sorted HSCs and Lin-cKit+Flk-1+ cells. The bars represent the fold change of Id1 mRNA levels in Lin-cKit+Flk-1+ cells relative to HSC levels. The results were normalized to HPRT expression and expressed as average fold change±SD (Lin-cKit+Flk-1+ 2.7±0.69 n = 4).

Id1-/- mice lack BM-derived circulating EPCs in the blood [5], [10]. Whether this is due to a defect in the generation of endothelial progenitors or to an inability of these cells to be released into the circulation is still unknown. We therefore examined the EPC population in the BM of wild type and Id1 mutant mice by analyzing the expression of cell surface markers previously used to detect blood circulating EPCs [10], [17], [21]. In wild type mice we identified Lin- cells that expressed both the endothelial marker Flk-1 and the progenitor marker c-Kit, representing about 0.2% of Lin- cells (Figure 2a) These cells were also largely positive (>80%) for VE-Cadherin, CD13 and AC133 (Figure 2b), characteristic markers of immature endothelial cells [22]. Notably, the Lin- Flk-1+ cKit+ population was severely depleted in the Id1-/- BM (less than 0.06% compared to 0.2% in wild type p<0.001) (Figure 2a). Consistent with a unique role in this cell subset, the level of Id1 mRNA in sorted Lin- Flk-1+ cKit+ cells is ∼2 fold higher than in uncommitted hematopoietic Lin-cKit+Sca-1+ (HSC) cells (fig 2d), previously shown to be relatively enriched for Id1 expression and functionally dependent on its activity [13]. To address the alternative possibility that the described Id1–dependent Lin- c-Kit+ Flk-1+ cells represent a previously uncharacterized subset of pro-angiogenic myeloid progenitors, we measured the relative expression of C/EBPα and PU.1, master regulators of granulocyte and monocyte differentiation, respectively [23], [24]. Both genes were expressed at a low level, comparable to the expression in uncommitted HSCs and inconsistent with a potential myeloid identity of the Lin- c-Kit+ Flk-1+ cells (Figure S2).

**Figure 3:**
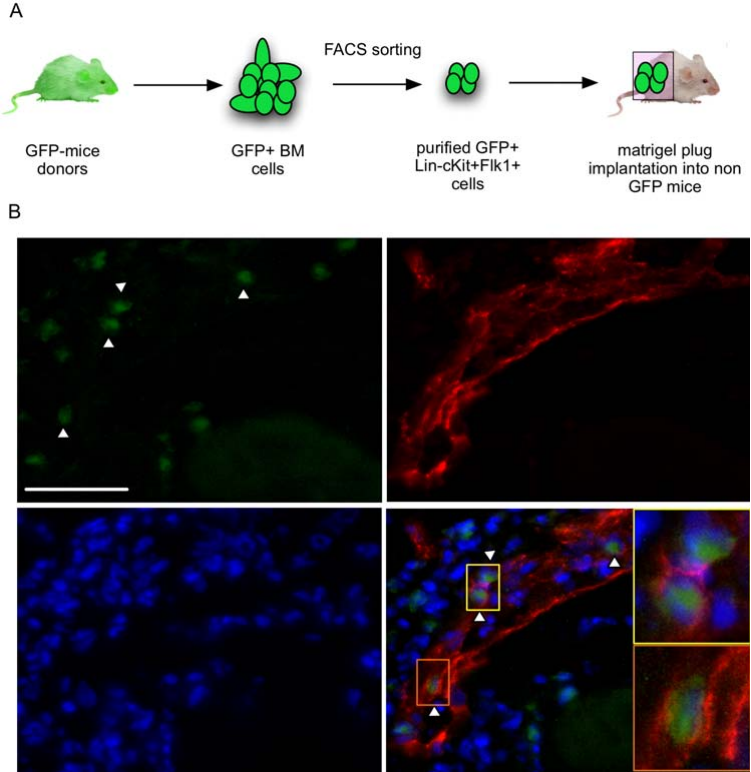
Lin- cKit+Flk-1+ BM cells have endothelial properties. A) Schematic representation of the experimental procedure. B) Immunofluorescence on matrigel sections showing the incorporation of GFP+ Lin- c-kit+ Flk-1+ cells into vessels (arrowheads). Yellow and orange insets show magnification of GFP+ cells. Green: GFP; Red: VE-Cadherin; Blue: DAPI. Scale bar 50 µm.

We have recently shown that Lin- VE-Cadherin+ Flk-1+ BM-derived cells differentiate into mature endothelial cells and incorporate into a vascular network when co-cultured with mature endothelial cells [9]. To assess *in vivo* whether Lin- c-Kit+ Flk-1+ BM cells have endothelial properties, we isolated this population from animals expressing the green fluorescent protein (GFP). Four hundred sorted cells were embedded into VEGF containing matrigel and injected subcutaneously into recipient mice (Figure 3a). Seven days post-implantation we observed VE-cadherin expressing GFP+ cells integrated into the vessels infiltrating the plug (Figure 3b). In addition, the number of GFP+ cells in the plug was dramatically increased relatively to the initial number of implanted cells, suggesting the ability of Lin- c-Kit+ Flk-1+ cells to proliferate in response to VEGF. These observations suggest that Lin- c-Kit+ Flk-1+ cells have properties of endothelial cells *in vivo* and that their loss may contribute to the defect in neo-angiogenesis described in the Id1 mutant mice.

### Loss of Id1 impairs the endothelial differentation of HSCs

**Figure 4:**
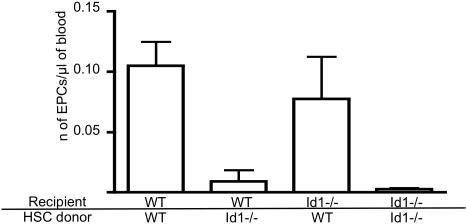
Id1 is necessary for the endothelial differentiation of BM stem cells. Flow cytometric analysis of EPCs in the peripheral blood of the indicated mice 4 weeks after transplantation of Lin- cKit+ Sca-1+ Flk-1- HSCs. The bars represent the average number or circulating EPCs (±SEM). per ml of blood. WT+WT HSCs: 0.105±0.002 (n = 3); WT+Id1-/- HSCs: 0.009±0.009 (n = 3); Id1-/-+WT HSCs: 0.077±0.034 (n = 4); Id1-/-+Id1-/- HSCs: 0.002±0.008 (n = 4).

The majority of Lin- cKit+ Flk-1+ cells do not express the stem cell marker Sca-1 (Figure 2c) suggesting that these cells do not belong to the pluripotent stem cell compartment [14] but rather could represent their more restricted progeny. It is well established that HSCs function as a source of endothelial cells in adult animals [15]–[17], demonstrating that during adulthood as well as during embryogenesis [25], [26] endothelial and hematopoietic cells represent alternative differentiation fates of a common progenitor. Since the absence of Id1 reduces the self-renewing capacity of the HSCs by promoting their commitment toward the myeloid lineage [13] we tested whether its absence could also reduce the multipotency of HSCs and affect their ability to differentiate into functional endothelial cells. To this end we purified Lin- cKit+ Sca-1+ HSCs [14] depleted of Flk-1+ cells from wild type and Id1-/- BM and used them to reconstitute lethally irradiated wild type and Id1-/- mice (Figure 4). Four weeks post-transplantation we observed a comparable number of circulating EPCs (defined as CD45- cKit+ CD13+ Flk-1+ cells [10], [21]) in the blood of wild type and Id1-/- recipient mice transplanted with wild type HSCs. In contrast, Id1-/- HSCs completely failed to reconstitute the endothelial population in either recipient, confirming that Id1 expression in HSCs is necessary to allow endothelial differentiation. The ability of wild type HSCs to reconstitute the EPC population in Id1-/- mice provides a possible explanation for the restored tumor angiogenesis previously observed in Id mutant mice after transplantation with wild type BM [5], [6], [10].

### Loss of Id1 does not affect the recruitment of pro-angiogenic hematopoietic cells to the tumor site

**Figure 5:**
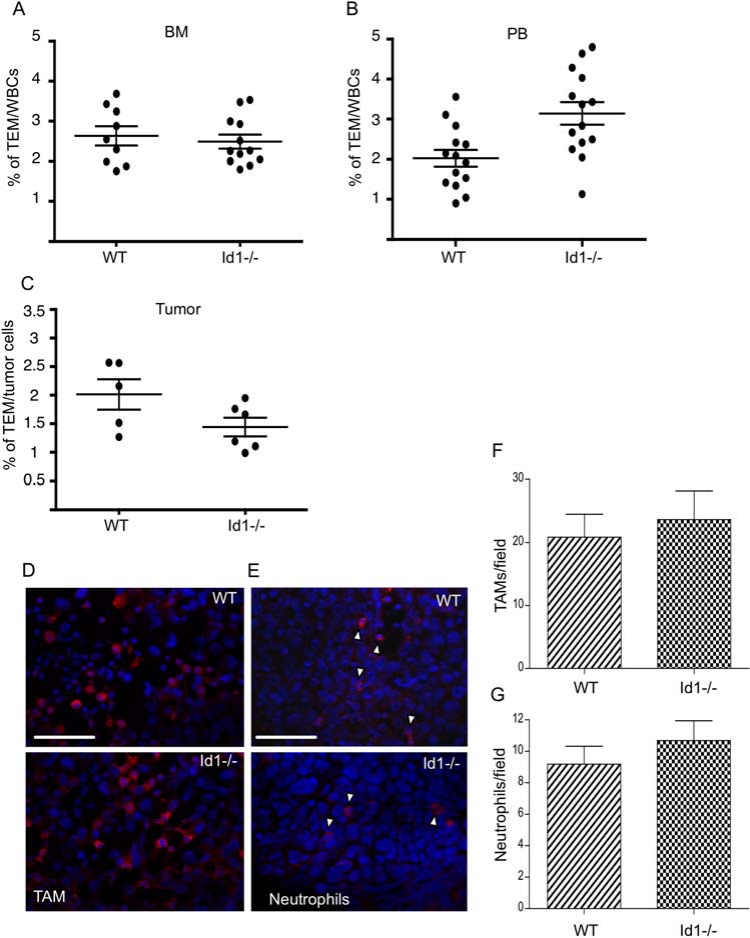
Loss of Id1 does not affect function of pro-angiogenic myeloid cells. Flow cytometry analysis of the TEMs in BM (A) peripheral blood (B) and LLC tumor (C) of wild type (wt) or Id1-/- mice. The plots represent the percentage of Tie2+ CD45+ Cd11b+ of nucleated cells in wild type and Id1-/- mice. Average±SEM: BM WT = 2.64±0.27 n = 9; Id1-/- = 2.5±0.17 n = 12. Peripheral blood WT = 2.1±0.21 n = 14; Id1-/- = 3.14±0.28 n = 14. Tumor WT = 2±0.27; Id1-/- = 1.44±0.16. D) Anti-CD68 immunofluorescence on LLC tumor sections showing infiltration of TAMs in WT and Id1-/- mice. Scale bar 50 µm. E) Anti-neutrophils antibody clone 7/4 immunofluorescence on LLC tumor sections showing infiltration of neutrophils in WT and Id1-/- mice. Scale bar 100 µm. F–G) Quantification of immunofluorescences shown in D and E. The histograms represent the number of positive cells counted per field (magnification 630X) in LLC tumors collected from WT and Id1-/- mice. A minimum of 6 non sequential sections were counted. TAM average±SEM (20.86±3.6 WT 23.67±4.5 Id1-/-) Neutrophils average±SEM (9.2±1.1 WT 10.7±1.2 Id1-/-).

A number of recent studies have emphasized the role of different hematopoietic cells in promoting tumor growth and angiogenesis [1]–[4]. Even though the analysis of BM-derived cells recruited into the tumor stroma indicates that Id1 expression is confined to the endothelial cells and is not detectable in other mature hematopoietic cells [6], [27] it is possible that a defect in other BM-derived cells contributes to the failure in angiogenesis observed in the Id1 mutant mice. In order to test this hypothesis we analyzed the ability of Id1-/- mice to recruit pro-angiogenic hematopoietic cells to growing tumors. Naldini and coworkers have identified a subset of myeloid cells (the Tie2 expressing monocyte or TEM) that are recruited to the tumor site and promote tumor angiogenesis in a paracrine manner [4]. We analyzed the frequency of TEMs in Id1-/- mice during tumor growth. Flow cytometry analysis showed that Id1-/- mice have a normal number of TEMs, defined as Tie2+ CD11b+ CD45+ cells [4] in both BM and peripheral blood (Figure 5a and b). Moreover, we observed no difference between wild type and Id1-/- mice in the recruitment of TEMs to the stroma of implanted tumors (Figure 5c), demonstrating that loss of Id1 does not affect the genesis, mobilization or function of Tie2 expressing monocytes. We also analyzed the infiltration of macrophages and neutrophils into tumors grown in Id1-/- mice. Using the tumor-associated macrophage marker CD68 [28] (Figure 5d) and the neutrophil marker clone 7/4 [29] (Figure 5e) we observed no difference between wild type and Id1-/- mice in the number or localization of positive cells infiltrating the tumor.

### Accelerated myeloid commitment of HSC cells in the absence of Id1 requires the expression of its target gene p21

**Figure 6:**
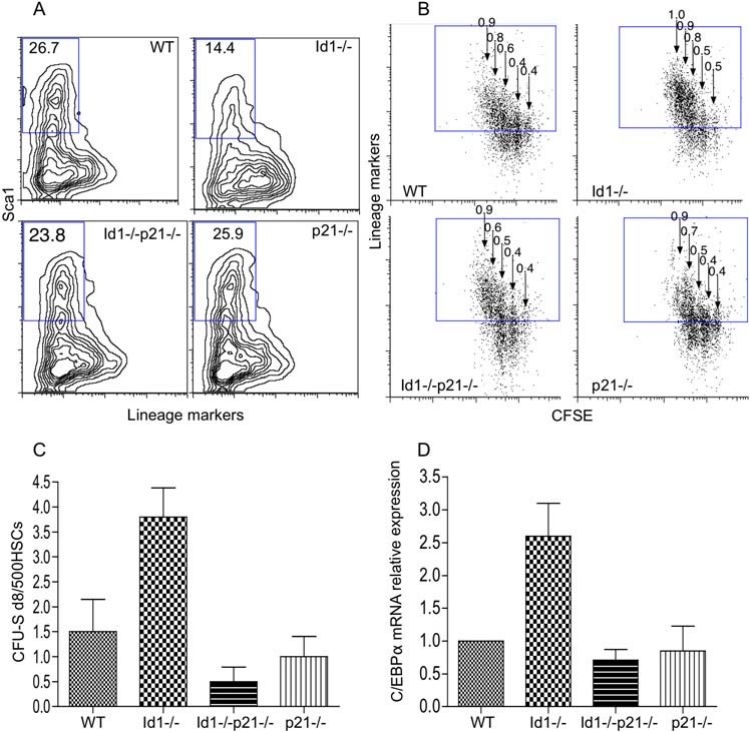
The increased myeloid priming of Id1 null HSCs requires the expression of p21 . A–B) In vitro differentiation of sorted HSCs in response to cytokine stimulation. We monitored the acquisition of hematopoietic lineage markers and the simultaneous loss of expression of Sca-1 (A). The numbers represent the percentage of Lin^low^ Sca-1^+^ cells remaining after 3 days in culture. The coupling of lineage differentiation with proliferation was analyzed by the progressive decrease in CFSE staining intensity with each cell division (B). The arrows correspond to each subsequent division, and the numbers represent the fraction of differentiated (Lin^+^) cells within each generation. The shown data is representative of three independent experiments. (C) Spleen colony forming assay using sorted Id1-/- HSCs cells. 500 sorted HSCs from the indicated groups of mice were transplanted into lethally irradiated recipients, and spleen colonies were scored on day 8 post-transplant. Numbers indicate average colony numbers±SEM (WT 1.5±0.64 n = 4; Id1-/- 3.8±0.58 n = 5; Id1-/-p21-/- 0.5±0.28 n = 4; p21-/- 1±0.40 n = 4) D) Quantitative real time PCR analysis of c/EBPα mRNA levels in sorted HSCs. The bars represent the fold change of c/EBPα mRNA levels in Id1-/-, Id1-/-p21-/- and p21-/- mice relative to WT levels. The results were normalized to HPRT expression and expressed as average fold change±SD (Id1-/- 2.6±0.7; Id1-/- p21-/- 0.71±0.22; p21-/- 0.85±0.53 n = 2).

The increased rate of myeloid commitment in the Id1-/- HSCs is associated with higher expression of the known target of Id1 repression, the cyclin-dependent kinase inhibitor p21 [13]. Previous studies have revealed a role for p21 in regulating lineage commitment of adult epidermal stem cells [30] and hematopoietic stem cells [31]. We reasoned that the increased level of p21 might contribute to the altered cell fate determination of Id1 null HSCs and restrict their potential to progress towards the endothelial lineage. To test this hypothesis we generated Id1-/-p21-/- mice and first analyzed whether ablation of p21 would counter the increased myeloid commitment rate of the Id1-/- HSCs. Sorted HSCs (Lin- cKit+ Sca1+ [14]) were cultured for 3 days in the presence of IL-3, IL-6 and SCF to promote early myeloid differentiation. Whereas Id1-/- HSCs showed accelerated expression of hematopoietic lineage markers and loss of the stem cell specific marker Sca-1 (as previously reported [13]) the Id1-/- p21-/- HSCs displayed a normal rate of differentiation (Figure 6a). To distinguish between an effect on cell differentiation versus proliferation, we stained sorted HSCs (Lin- cKit+ Sca1+ [14]) with the vital dye CFSE [32] to track the expression of lineage markers for each cell division. The Id1-/- HSCs undergo more divisions, and within each generation show an increased expression of the lineage markers compared to wild type HSCs (Figure 6b). The ablation of p21 in Id1-/- HSCs completely reverses the effects of Id1 loss on both the proliferation and differentiation dynamics. In contrast, no significant effect of p21 loss is observed in Id1 wild type cells, arguing against Id1–independent effects of p21 deletion on cell cycle regulation in this assay. Furthermore, the increased *in vivo* spleen colony formation by Id1 HSCs 8 days post-transplant (CFU-S8), characteristic of the premature myelo-erythroid commitment [13], was also completely corrected in Id1-/-p21-/- HSCs (Fig 6c).

As previously reported, Id1-/- HSCs have increased expression of myeloid specific genes including the transcription factor c/EBPalpha [13], a critical regulator of myeloid commitment in HSCs [33]. Loss of p21 abolishes the upregulation of c/EBPα mRNA in the Id1-/- HSCs (Figure 6c). This suggests that higher p21 levels could actually promote myeloid differentiation of Id1-/-HSCs.

### Ablation of p21 restores a normal Lin- Flk-1+ cKit+ population in the Id1 mutant mice

**Figure 7:**
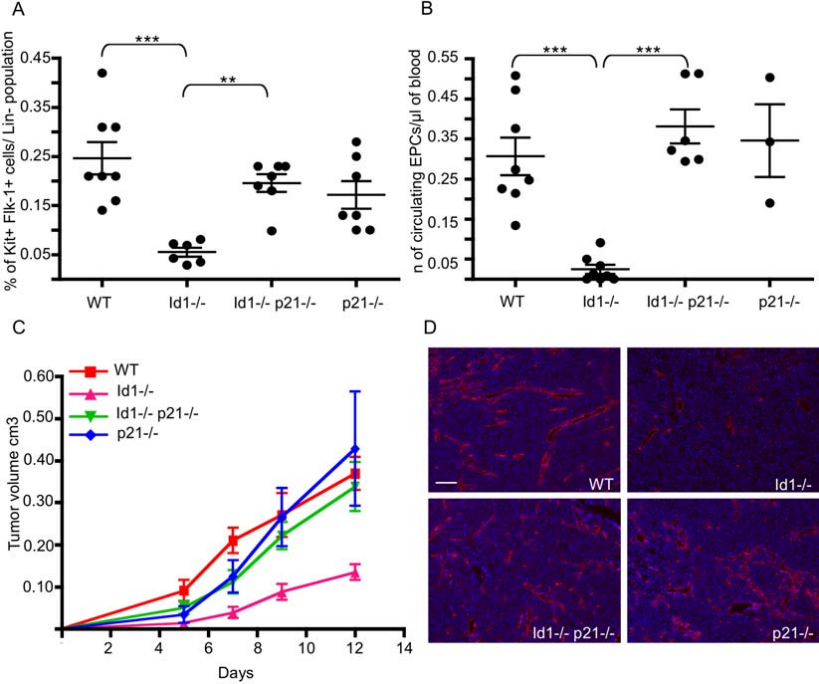
Loss of p21 rescues functional BM-derived tumor angiogenesis in Id1-/- mice. A) Flow cytometric analysis of the EPC fraction of BM cells from wild type; Id1-/-; Id1-/- p21-/- and p21-/- mice. The plots represent the percentage of cKit+ Flk-1+ cells in the population of Lin- BM cells in the indicated groups of mice. Average±SEM : wt = 0.25±0.03 (n = 8); Id1-/- = 0.054±0.009 (n = 6); Id1-/- p21-/- = 0.2±0.018 (n = 7); p21-/- = 0.17±0.02 (n = 7). p = 0.0007 ***, p = 0.0012 ** B) Flow cytometry analysis of the circulating EPC fraction in the peripheral blood of wt; Id1-/-; Id1-/- p21-/- and p21-/- mice. The plots represent the number of CD45-cKit+ Flk-1+ CD13+ cells per µl of blood in the different groups of mice. Average±SEM: wt = 0.31±0.04 (n = 8); Id1-/- = 0.024±0.01 (n = 8); Id1-/- p21-/- = 0.38±0.042 (n = 6); p21-/- = 0.35±0.09 (n = 3). p<0.001 *** C) Growth curve of LLC tumors implanted into wild type; Id1-/-; Id1-/- p21-/- and p21-/- mice. D) VE-cadherin immunofluorescence on tumor sections showing the vascular density in the indicated genotypes. Scale bar 100 µm.

To assess whether ablation of p21 can also restore the angiogenic potential of Id1-/- BM cells, we analyzed the frequency of Lin- Flk-1+ cKit+ by flow cytometry in wild type, Id1-/-, p21-/- and Id1-/- p21-/- mice (Figure 7a). The severe deficiency of these cells observed in the Id1-/- mice was nearly completely corrected in the Id1-/- p21-/- mice. Furthermore we detected a comparable number of circulating EPCs [10], [21] in the blood of wild-type and Id1-/-p21-/- double mutant mice (Figure 7b), while almost none were detectable in the blood of Id1-/- mice, as previously reported [10]. Moreover, flow cytometric analysis of tumor tissue showed a comparable number of infiltrating CD11b- VE-Cadherin+ cells in wild type and Id1-/-p21-/- mice (data not shown). To demonstrate that ablation of p21 rescues the ability of Id1-/- HSCs to generate the endothelial population we transplanted 4 groups of sublethally irradiated Id1-/- mice with 20 highly purified long term repopulating HSCs (defined as Lin- cKit+ Sca1+ and CD34-) [34] depleted of any Flk-1+ cells, isolated by flow cytometry from wild type, Id1-/-, Id1-/-p21-/- and p21-/- mice. Four weeks after transplantation we analyzed the frequency of circulating EPCs by flow cytometry. While no EPCs were detected in the blood of Id1-/- mice transplanted with Id1-/- HSCs, the Id1-/-p21-/- HSCs generated endothelial progeny in the blood of the recipient mice. The frequency of HSC-derived circulating EPCs was significantly lower in the recipients of both p21-/- and Id1-/-p21-/- than wild-type HSCs, indicating additional, Id1-independent effects of p21 loss on circulating EPC differentiation and/or homeostasis. (Figure S3.)

To demonstrate that the Id1-/- p21-/- Lin- Flk-1+ cKit+ cells were functionally able to sustain tumor angiogenesis we analyzed the rate of tumor growth in the Id1-/- p21-/- mice. 2×10^6^ Lewis Lung Carcinoma (LLC) tumor cells were injected subcutaneously into wild type, Id1-/-, p21-/- and Id1-/-p21-/- mice and tumor growth followed for 12 days (Figure 7c). As previously reported [5], [35], Id1-/- mice showed a slower rate of tumor growth compared to wild type mice (80% less growth seen 7 days after implantation p<0.001). However, a similar tumor growth rate was observed in wild type, p21-/- and Id1-/- p21-/- mice throughout the duration of the experiment. Since slower tumor growth in the Id1 mutant mice has been associated with decreased density and abnormal structure of the vasculature [35], we analyzed the vascular organization of the LLC tumors grown in the four different groups of mice (Figure 7d and Figure S4). Staining for VE-cadherin expression showed a homogeneous density of large vessels and small capillaries invading the stroma of tumors grown in wild type mice. As expected, tumors grown in the Id1-/- mice displayed a significant decrease in the number and size of the vessels. In accordance with the restored tumor growth observed in the Id1-/- p21-/- mice, the tumors taken from these animals showed a normal vascular density. Moreover, transplantation of Id1-/- p21-/- BM into Id1 mutant mice rescued normal tumor growth (data not shown) further confirming that the correction of the angiogenic phenotype observed in the double mutant mice is due to the effects in the BM compartment and not to effects on other peripheral components of the tumor stroma. Overall these observations support the hypothesis that the defect in BM-derived tumor angiogenesis described in the Id1 mutant mice is due primarily to a differentiation failure within the endothelial lineage. However, we cannot exclude the possibility that normalization of other BM populations might also contribute to the rescue observed in the Id1/p21 double mutant mice.

## Discussion

It has been known for several years that depletion of the dominant negative helix-loop-helix protein Id1 in mice leads to a failure of tumor angiogenesis and consequently a significant delay in tumor growth [5], [10]. While a role for Id1 in the non-BM derived endothelial cell component of some tumors has been established [6], in other settings it is clear that the phenotype observed in the Id1-/- mice is due to a failure in the BM compartment since normal tumor angiogenesis can be completely rescued by transplantation with wild type BM [5], [6], [10]. We have previously shown that loss of Id1 leads to the absence of BM-derived blood circulating EPCs in both the resting state and after pro-angiogenic stimulation [5]–[10]. Two general models, not necessarily mutually exclusive, could be imagined: progenitor cells are formed in the BM but fail to migrate out into the blood circulation or the cells fail to form in the first place. The experiments described here demonstrate that in Id1 mutant BM there is profound reduction in a population of cells (Lin- Flk-1+ cKit+) that have properties of EPCs. This population expresses characteristic progenitor cell markers (i.e. Lin-, ckit+ AC133 +) and endothelial cell markers (VEGFR2+, VE-Cadherin+, CD13+) (Figure 2) and has a pattern of gene expression different from myeloid cells (Figure 2 and Figure S2). In addition, when purified these cells have the ability to respond *in vivo* to VEGF and become incorporated into mature vessels (Figure 3). In humans, the stem cell markers CD34 and CD133 have been used together to identify endothelial progenitor cells in clinical samples but cannot be used in the mouse owing to the lack of CD34 expression on EPCs [22]. While we cannot rule out the possibility that in the mouse other BM cell types are affected by loss of Id1, our results argue strongly that the absence of the endothelial progenitor population defined here is a critical component of the angiogenic defect observed in the Id1-/- mice. Indeed we show that while the Lin- Flk-1+ cKit+ population is profoundly depleted, three major BM-derived pro-angiogenic hematopoietic cell types (Tie2 expressing monocytes, tumor associated macrophages and neutrophils) are normal in the absence of Id1 (Figure 5). Moreover, restoration of Lin- Flk-1+ cKit+ cells in the BM by genetic manipulation (see below) leads to a complete reversal of the Id1 null defective vascular phenotype.

How does Id1 control the formation of these cells in the BM? We have reported previously that the loss of Id1 leads to the premature commitment of hematopoietic stem cells to the myeloid lineage [13] and we demonstrate here that this loss also affects the formation of the endothelial population (Figure 4). Several studies in the past decade have demonstrated that adult HSCs can give rise to functional endothelial cells [15]–[17] and we now demonstrate that Id1 expression is critical for this process.

Upregulation of the cyclin dependent kinase inhibitor p21 as a result of Id1 loss is associated with enhanced cycling of the stem cell population, accelerated expression of myeloid markers and loss of EPCs. Ablation of p21 in the Id1 mutant background (through genetic manipulation) completely corrects the differentiation defect of HSCs restoring a normal EPC population in the BM and in the peripheral blood and rescuing normal tumor angiogenesis (Figure 6 and 7). Under steady-state conditions the expression of p21 is enriched in HSCs relative to committed hematopoietic progenitors [36], maintaining the stem cell self-renewal capacity perhaps by regulating their cell cycle entry [37]. Despite its growth inhibitory function in HSCs, several studies have demonstrated a positive effect of p21 induction on growth and differentiation in transiently amplifying myeloid progenitors [38], [39]. Consistent with this, the proliferation and differentiation of G-CSF mobilized murine HSCs is associated with a relative induction of p21 [40], indicating a possible positive role in the HSC differentiation program. We propose that increased p21 levels in the absence of Id1 could act similarly and support the increased myeloid lineage commitment of Id1-/- HSCs. Although further study is required to dissect the molecular mechanisms by which Id1 and p21 regulate HSC commitment, we suppose that upregulation of p21 (induced by loss of Id1) not only primes HSCs toward the myeloid fate but that premature priming may deplete the endothelial progenitor cell population. It is interesting to note that by inhibiting the formation of EPCs, p21 may act as a tumor suppressor by both inhibiting tumor angiogenesis (by repressing EPCs formation in the BM) as well as by restraining tumor cell proliferation directly.

The upregulation of p21 in response to Id1 loss may be a direct or indirect consequence of the liberation of E proteins. Indeed, in fibroblasts overexpression of E proteins leads to the direct activation of the p21 promoter [41] but in HSCs the situation may be more complex. It is also not yet clear how p21 upregulation, in the absence of Id1, leads to the coordinated skewing of gene expression towards that of the myeloid lineage. Nevertheless, the observed effect of p21 induction is not consistent with its canonical cell cycle inhibitory role, as cultured Id1 null HSCs undergo more proliferation than wild type cells, perhaps due to the pro-proliferative activity of p21 that has been reported in other systems [42].

As has been pointed out previously [43] it is unlikely that the Id proteins are “hard-wired” to drive the progression of the cell cycle as Id1, 2, 3 triple knockout embryos undergo many rounds of cell division prior to embryonic lethality, suggesting an effect on cell fate which may in some cases impinge on cell cycle progression. In fact, loss of Id1 leads to enhanced proliferation of HSCs both in vitro as well as in vivo [13]. However, proliferation per se is not the sole reason for the altered differentiation profile in the Id1 knockout cells since vital dye marking of the HSCs, which allows for marker analysis at each cell division, shows a clear change in differentiation status at each cell division.

The extent of utilization of BM derived endothelial cells has been shown to vary with tumor type and tumor grade [6], [7]. Even under circumstances where low incorporation of these cells is observed, their importance has been supported by bone marrow transplantion studies using the Id1 mutant model in which the EPC function is completely impaired [5], [6], [10]. We have shown recently that utilization of EPCs is most prominent early in tumor development and that antibody ablation specifically of the EPC population mimics the phenotype described in the Id1-/-mice with abnormal tumor vasculature and delayed tumor growth [8], [9]. Other BM derived cell types have also been shown to be important in the neoangiogenesis process [1]–[4] however, the relative contribution of each remains a subject of intense debate. While this issue is not resolved fully by these studies, our work supports the idea that Id1-expressing BM-derived EPCs are a critical component of tumor neovascularization: Id1 is required for normal tumor angiogenesis, Id1 expression in HSCs is required for the generation of progenitor cells with the ability to become incorporated into blood vessels, Id1 is only expressed in tumor endothelial cells and not macrophages or pericytes [6], and Id1 loss does not affect the levels of monocytes, macrophages or neutrophils in the peripheral blood or at the site of the tumor (Figure 5).

In conclusion, this work describes for the first time the opposing activity of two regulators of the differentiation of BM-derived EPCs from BM stem cells, Id1 and p21, and supports the importance of these cells during tumor angiogenesis. Further studies will be required to fully characterize the regulatory mechanisms that govern the function of these cells and therefore to design functional strategies to inhibit their activity.

## Materials and Methods

### Animal and tumor models

All studies were conducted using age matched (8–10 week old) C57B6/Sv129 mice. Actin-GFP transgenic mice C57BL/6-Tg(ACTbEGFP)10sb/J were purchased from The Jackson Laboratory (Bar Harbor, Maine). The animals were maintained in pressurized ventilated caging at MSKCC according to IACUC approved protocol. Lewis Lung Carcinoma cells (LLC) (ATCC, Manassas, VA) were cultured in DMEM supplemented with 10% FBS. For tumor growth experiments, 2×10^6^ LLC cells were injected subcutaneously into recipient mice. Tumor size was assessed every two days using a Venier caliper. After 14 days, tumors were surgically removed and analyzed by immunofluorescence. The AdVEGF165 and AdNull vectors were previously described [5] and obtained from Dr. Ronald Crystal. Mice were injected intravenously with 1.5×10^8^ particle forming units (pfu) of AdVEGF165 and AdNull. 48 hours after injection the animals were sacrificed and femurs collected for histological analysis. For the BM transplantation experiments, age matched wild type and Id1-/- mice were lethally irradiated (10 Gy) and reconstituted with 5000 sorted Lin- cKit+ Sca-1+ Flk-1- HSCs, along with 5×10^5^ syngenic Id1-/- total BM mononuclear cells. The Flk-1- HSCs mice used for transplantation were sorted as described below, from age matched wild type and Id1-/- mice. For the matrigel experiments, GFP+Lin- cKit+ Flk-1+ BM-EPCs were sorted as described below. 400 sorted cells were resuspended in 400 µl of grow factor reduced matrigel (BD Bioscences) supplemented with 400ng/ml of mouse VEGF (Peprotech, NJ) and injected subcutaneously into recipient mice. Seven days after implantation the matrigel plug was removed and prepared for histological analysis, as described below.

### Immunohystochemistry and Immunofluorescence

Femurs were fixed in 10% formalin for 24 hours, decalcified in EDTA/Formalin for additional 24 hours embedded into paraffin and sectioned. 5 µm sections were stained with a rabbit monoclonal antibody against mouse Id1 [27], using the ABC kit (Vector Laboratories) according to the manufacturer's instruction. Tumors and matrigel plugs were removed and immediately embedded in Optimal Cutting Temperature (O.C.T.) compound (Sakura Finetek, CA). 8 µm sections were stained with rat anti-VE-cadherin (BD Pharmingen), rat anti-CD68 [28] and rat anti- neutrophil clone 7/4 [29] (Serotec) followed by staining with Alexa 546-conjugated goat anti-rat antibody (Molecular Probe). Images were captured using an Axiovert 200 microscope (Zeiss).

### Antibodies and Flow Cytometry

For flow cytometry analysis and sorting, we used the following antibodies: Biotin conjugated anti mouse lineage cocktail (containing CD3, CD4, CD5, CD8, B220, Gr1, CD11b, Ter119 antibodies), PercP conjugated anti Streptavidin, APC-Cy7- conjugated anti Streptavidin, APC-conjugated anti c-Kit, PE-conjugated anti Flk-1, Sca-1 and CD45, FITC-conjugated anti CD13, Sca-1 and Cd11b, PerCP conjugated CD45 PerCP Alexa 647-conjugated VE-cadherin (BD Pharmingen); FITC-conjugated AC133 and PE-conjugated Tie2 (e-Bioscence, CA); 7AAD (Calbiochem). BM cells were collected from femurs and tibiae in X-VIVO 20 media (Cambrex, CA) for analysis and sorting and stained with the indicated combination of antibody. Flow cytometry analysis of blood circulating EPCs was carried out as previously described [10], [23] , as was the analysis of TEMs in the BM and blood reported [4]. Tumors were digested at 37°C for 45–60 min with an enzyme cocktail (Collagenase A, elastase, and DNase I, Roche Applied Science) and filtered through a 30-um strainer. A single cell suspension was stained to identify TEMs as previously report [4]. Flow cytometry analysis was performed using the FACSCalibur cytometer (Becton Dickinson, CA). Flow cytometry sorting was performed using the MoFlo cell sorter from Cytomation, (Fort Collins, CO). All flow cytometry data were analyzed using FloJo software package (Tree Star, Mountain View, CA).

### Analysis of in vitro HSC myeloid differentiation

HSCs were sorted by flow-cytometry as described above. Sorted cells were seeded at a density of 5000cells/well in the non-tissue culture treated 12-well plates (BD Falcon, CA), with 1 ml of culture media. For all *in vitro* culture we used X-vivo 15 serum-free media (Cambrex, CA), supplemented with 55 µM 2-mercaptoethanol (Invitrogen) and a cocktail of early-acting myeloid supporting cytokines −100 ng/ml SCF , 10 ng/ml IL-3 and 10 ng/ml IL-6 (Peprotech, NJ). For the in vitro differentiation assays, cells were analyzed for lineage markers and Sca-1 expression by flow-cytometry following 72h of culture. For the in vitro cell division tracking experiments, cells were labeled with a vital dye CFSE (carboxyfluoroscein succinimidyl ester) (Molecular Probes) prior to seeding, as previously described [32]. Following 72 h of culture, cells were simultaneously analyzed for lineage marker expression and CFSE fluorescence. The discrete peaks of CFSE fluorescence corresponding to the individual generations of divided cells, were determined using the proliferation analysis module of the FloJo software package (Tree Star, Mountain View, CA). For the in vivo spleen colony forming assay, lethally irradiated recipients were transplanted with 500 sorted Lin- cKit+ Sca1+ cells/mouse. Transplant recipients were sacrificed and macroscopic spleen colonies were scored 8 days post-transplant.

### Quantitative Real Time PCR

BM Lin- progenitors and HSCs used for qRT-PCR were sorted as described above. Total RNA was collected using RNAeasy kit (QIAGEN, CA), (for HSCs only) amplified using Gene Chip Two-Cycle cDNA Synthesis (Affimetryx, CA) and retrotranscribed using ThermoScript RT-PCR System (Invitrogen). Quantitative PCR was carried out using the SYBR green PCR Mastermix (Applied Biosystems) and the 7900 Real Time PCR system (Apllied Biosystems). Relative expression of target genes was calculated using standard curve method and normalized to HPRT mRNA content. Primers used: Id1 Forward 5′-CGA CTA CAT CAG GGA CCT GCA-3′ Id1 Reverse 5′-GAA CAC ATG CCG CCT CGG-3′; cEBP alpha Forward 5′-GAT CTG CGA GCA CGA GAC-3′ cEBP alpha Reverse 5′-CTT GGC CTT CTC CTG CTG-3′.

## Supporting Information

Figure S1 Id1 is upregulated in Lin- progenitor cells upon pro-angiogenic stimuli. A) Quantitative real time PCR analysis of Id1 mRNA levels in Lin- sorted BM cells from wild type mice untreated or 4 days after LLC tumor implantation. The bars represent fold induction of Id1 mRNA levels in sorted Lin- cells upon tumor implantation relative to steady state levels. The results were normalized to HPRT expression and expressed as average fold induction±SD (4.84±0.45; n = 3). B) Western blot analysis of Id1 protein (upper panel) and actin (lower panel) in sorted Lin- cells from wild type mice untreated or 4 days after LLC tumor implantation.
(0.15 MB TIF)

Figure S2 Lin- cKit+ Flk-1+ cells do not show higher expression of myeloid determining genes compared to incommitted HSCs. Quantitative real time PCR analysis of c/EBPα (A) and PU.1 (B) mRNA levels in sorted HSCs and Lin-cKit+Flk-1+ cells. The bars represent the fold change of c/EBPα and PU.1 mRNA levels in Lin-cKit+Flk-1+ cells relative to HSC levels. The results were normalized to HPRT expression and expressed as average fold change±SD (c/EBPα 1.3±0.17 n = 6; PU.1 1.3±0.147 n = 6).
(0.26 MB TIF)

Figure S3 Ablation of p21 rescues the endothelial differentiation defect of Id1-/- HSCs. Id1-/- mice were sublethally irradiated then transplanted with 20 Lin- cKit+ Sca-1+ CD34- Flk-1- HSCs purified from the BM of the indicated group of mice. The histograms represent the flow cytometry analysis of circulating EPCs in the peripheral blood of Id1-/- mice 4 weeks after transplantation. The bars represent the average number or circulating EPCs (±SEM) per µl of blood. WT HSCs: 0.4±0.007 (n = 5); Id1-/- HSCs: 0.0001±0.0003 (n = 3); Id1-/-p21-/- HSCs: 0.06
(0.13 MB TIF)

Figure S4 Quantification of vessels in LLC tumors from WT, Id1-/-, Id1-/-p21-/- and p21-/- mice. A minimum 400 vessels were counted from 5 non sequential sections were counted. Average±SEM: WT 51±8.8; Id1-/- 14.56±1.9; Id1-/-p21-/- 48.78±5.7 and p21-/- 41.89±3.55.
(0.12 MB TIF)

## References

[1] Coussens LM, Raymond WW, Bergers G, Laig-Webster M, Behrendtsen O (1999). Inflammatory mast cells up-regulate angiogenesis during squamous epithelial carcinogenesis.. Genes Dev.

[2] Balkwill F, Charles KA, Mantovani A (2005). Smoldering and polarized inflammation in the initiation and promotion of malignant disease.. Cancer Cell.

[3] Coukos G, Benencia F, Buckanovich RJ, Conejo-Garcia JR (2005). The role of dendritic cell precursors in tumour vasculogenesis.. Br J Cancer.

[4] De Palma M, Venneri MA, Galli R, Sergi Sergi L, Politi LS (2005). Tie2 identifies a hematopoietic lineage of proangiogenic monocytes required for tumor vessel formation and a mesenchymal population of pericyte progenitors.. Cancer Cell.

[5] Lyden D, Hattori K, Dias S, Costa C, Blaikie P (2001). Impaired recruitment of bone-marrow-derived endothelial and hematopoietic precursor cells blocks tumor angiogenesis and growth.. Nat Med.

[6] Ruzinova MB, Schoer RA, Gerald W, Egan JE, Pandolfi PP (2003). Effect of angiogenesis inhibition by Id loss and the contribution of bone-marrow-derived endothelial cells in spontaneous murine tumors.. Cancer Cell.

[7] Peters BA, Diaz LA, Polyak K, Meszler L, Romans K (2005). Contribution of bone marrow-derived endothelial cells to human tumor vasculature.. Nat Med..

[8] Singh Jaggi J, Henke E, Seshan SV, Kappel BJ, Chattopadhyay D (2007). Selective alpha-particle mediated depletion of tumor vasculature with vascular normalization.. PLos ONE.

[9] Nolan DJ, Ciarrocchi A, Mellick AS, Jaggi JS, Bambino K (2007). Bone marrow derived endothelial progenitor cells are a major determinant of nascent tumor neovascularization.. Genes Dev.

[10] Shaked Y, Ciarrocchi A, Franco M, Lee CR, Man S (2006). Therapy-induced acute recruitment of circulating endothelial progenitor cells to tumors.. Science.

[11] Ruzinova MB, Benezra R (2003). Id proteins in development, cell cycle and cancer.. Trends Cell Biol.

[12] Perk J, Iavarone A, Benezra R (2005). Id family of helix-loop-helix proteins in cancer.. Nature Rev. Cancer.

[13] Jankovic V, Ciarrocchi A, Boccuni P, Deblasi T, Benezra R (2007). Id1 restrains myeloid commitment, maintaining the self-renewal capacity of hematopoietic stem cells.. Proc Natl Acad Sci U S A.

[14] Morrison SJ, Weissman IL (1994). The long-term repopulating subset of hematopoietic stem cells is deterministic and isolatable by phenotype.. Immunity.

[15] Grant MB, May WS, Caballero S, Brown GA, Guthrie SM (2002). Adult hematopoietic stem cells provide functional hemangioblast activity during retinal neovascularization.. Nat Med.

[16] Bailey AS, Jiang S, Afentoulis M, Baumann CI, Schroeder DA (2004). Transplanted adult hematopoietic stems cells differentiate into functional endothelial cells.. Blood.

[17] Sebzda E, Hibbard C, Sweeney S, Abtahian F, Bezman N (2006). Syk and Slp-76 mutant mice reveal a cell-autonomous hematopoietic cell contribution to vascular development.. Dev Cell.

[18] Calvi LM, Adams GB, Weibrecht KW, Weber JM, Olson DP (2003). Osteoblastic cells regulate the haematopoietic stem cell niche.. Nature.

[19] Zhang J, Niu C, Ye L, Huang H, He X (2003). Identification of the haematopoietic stem cell niche and control of the niche size.. Nature.

[20] Kiel MJ, Yilmaz OH, Iwashita T, Yilmaz OH, Terhorst C (2005). SLAM family receptors distinguish hematopoietic stem and progenitor cells and reveal endothelial niches for stem cells.. Cell.

[21] Bertolini F, Paul S, Mancuso P, Monestiroli S, Gobbi A (2003). Maximum tolerable dose and low-dose metronomic chemotherapy have opposite effects on the mobilization and viability of circulating endothelial progenitor cells.. Cancer Res.

[22] Bertolini F, Shaked Y, Mancuso P, Kerbel RS (2006). The multifaceted circulating endothelial cell in cancer: towards marker and target identification.. Nat Rev Cancer.

[23] Zhang DE, Hetherington CJ, Chen HM, Tenen DG (1994). The macrophage transcription factor PU.1 directs tissue-specific expression of the macrophage colony-stimulating factor receptor.. Mol. Cell Biol..

[24] Zhang DE, Zhang P, Wang ND, Hetherington CJ, Darlington GJ (1997). Absence of granulocyte colony-forming factor signaling and neutrophil development CCAAT enhancer binding protein alpha-deficient mice.. Proc. Nat. Acad. Sci. USA.

[25] Shalaby F, Rossant J, Yamaguchi TP, Gertsenstein M, Wu XF (1995). Failure of blood-island formation and vasculogenesis in Flk-1-deficient mice.. Nature.

[26] Kennedy M, Firpo M, Choi K, Wall C, Robertson S (1997). A common precursor for primitive erythropoiesis and definitive haematopoiesis.. Nature.

[27] Perk J, Gil-Bazo I, Chin Y, de Candia P, Chen JJ (2006). Reassessment of id1 protein expression in human mammary, prostate, and bladder cancers using a monospecific rabbit monoclonal anti-id1 antibody.. Cancer Res.

[28] Nozawa H, Chiu C, Hanahan D (2006). Infiltrating neutrophils mediate the initial angiogenic switch in a mouse model of multistage carcinogenesis.. Proc Natl Acad Sci U S A.

[29] Hirsch S, Gordon S (1983). Polymorphic expression of a neutrophil differentiation antigen revealed by monoclonal antibody 7/4.. Immunogenetics.

[30] Topley GI, Okuyama R, Gonzales JG, Conti C, Dotto GP (1999). p21(WAF1/Cip1) functions as a suppressor of malignant skin tumor formation and a determinant of keratinocyte stem-cell potential.. Proc Natl Acad Sci U S A.

[31] Kirstetter P, Anderson K, Porse BT, Jacobsen SE, Nerlov C (2006). Activation of the canonical Wnt pathway leads to loss of hematopoietic stem cell repopulation and multilineage differentiation block.. Nat Immunol.

[32] Lyons AB, Parish CR (1994). Determination of lymphocyte division by flow cytometry.. J Immunol Methods.

[33] Zhang P, Iwasaki-Arai J, Iwasaki H, Fenyus ML, Dayaram T (2004). Enhancement of hematopoietic stem cell repopulating capacity and self-renewal in the absence of the transcription factor C/EBP alpha.. Immunity.

[34] Osawa M, Hanada K, Hamada H, Nakauchi H (1996). Long-term lymphohematopoietic reconstitution by a single CD34-low/negative hematopoietic stem cells.. Science.

[35] Lyden D, Young AZ, Zagzag D, Yan W, Gerald W (1999). Id1 and Id3 are required for neurogenesis, angiogenesis and vascularization of tumour xenografts.. Nature.

[36] Forsberg EC, Prohaska SS, Katzman S, Heffner GC, Stuart JM (2005). Differential Expression of Novel Potential Regulators in Hematopoietic Stem Cells.. PLoS Genet.

[37] Cheng T, Rodrigues N, Shen H, Yang Y, Dombkowski D (2000). Hematopoietic stem cell quiescence maintained by p21cip1/waf1.. Science.

[38] Braun SE, Mantel C, Rosenthal M, Cooper S, Liu L (1998). A positive effect of p21cip1/waf1 in the colony formation from murine myeloid progenitor cells as assessed by retroviral-mediated gene transfer.. Blood Cells Mol Dis.

[39] Yaroslavskiy B, Watkins S, Donnenberg AD, Patton TJ, Steinman RA (1999). Subcellular and cell-cycle expression profiles of CDK-inhibitors in normal differentiating myeloid cells.. Blood.

[40] Passegue E, Wagers AJ, Giuriato S, Anderson WC, Weissman IL (2005). Global analysis of proliferation and cell cycle gene expression in the regulation of hematopoietic stem and progenitor cell fates.. J Exp Med.

[41] Prabhu SIA, Park ST, Sun XH (1997). Regulation of the Expression of Cyclin-Dependent Kinase Inhbitor p21by E2A and Id1 proteins.. Molecular and Cellular Biology.

[42] Dong Y, Chi SL, Borowsky AD, Fan Y, Weiss RH (2004). Cytosolic p21Waf1/Cip1 increases cell cycle transit in vascular smooth muscle cells.. Cell Signal.

[43] Fraidenraich D, Stillwell E, Romero E, Wilkes D, Manova K (2004). Rescue of cardiac defects in id knockout embryos by injection of embryonic stem cells.. Science.

