# Supplementary figures and images for: Id1 Restrains p21 Expression to Control Endothelial Progenitor Cell Formation
Source: PLoS One. 2007 Dec 19;2(12):e1338. doi: 10.1371/journal.pone.0001338 (PMC2129121; doi:10.1371/journal.pone.0001338)

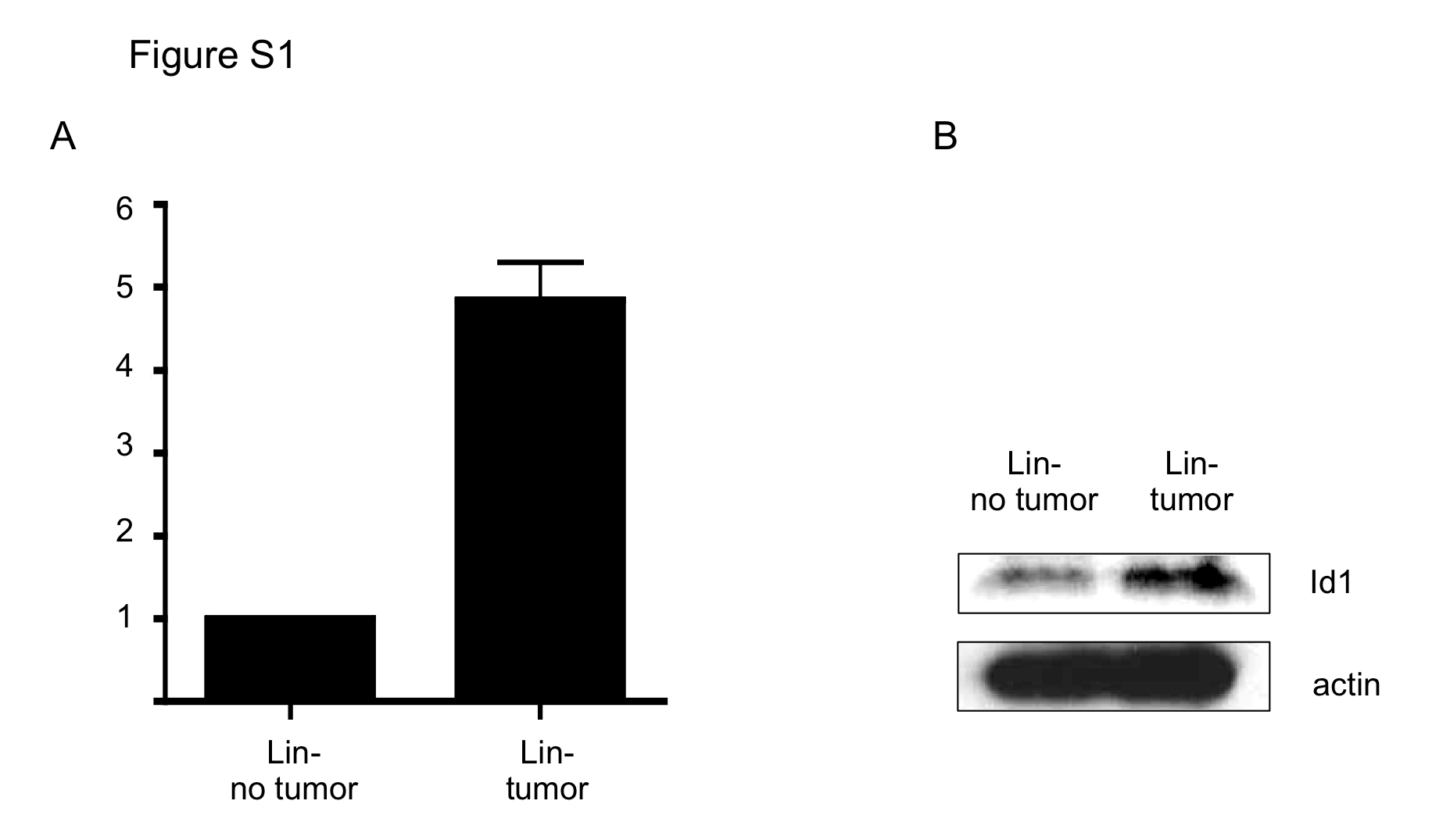

Supplement: Figure S1 — Id1 is upregulated in Lin- progenitor cells upon pro-angiogenic stimuli. A) Quantitative real time PCR analysis of Id1 mRNA levels in Lin- sorted BM cells from wild type mice untreated or 4 days after LLC tumor implantation. The bars represent fold induction of Id1 mRNA levels in sorted Lin- cells upon tumor implantation relative to steady state levels. The results were normalized to HPRT expression and expressed as average fold induction±SD (4.84±0.45; n = 3). B) Western blot analysis of Id1 protein (upper panel) and actin (lower panel) in sorted Lin- cells from wild type mice untreated or 4 days after LLC tumor implantation. (0.15 MB TIF) [file pone.0001338.s001.tif]

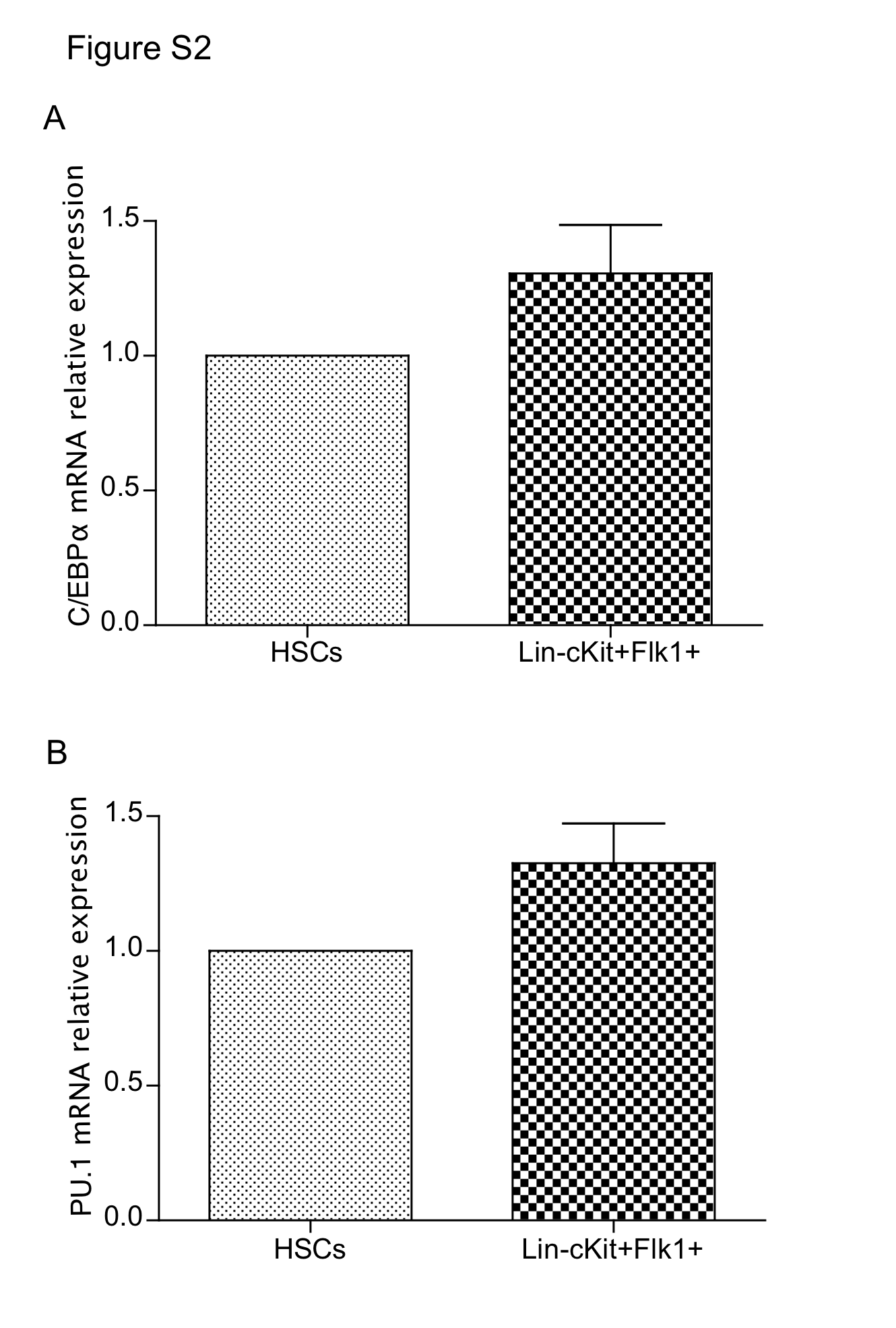

Supplement: Figure S2 — Lin- cKit+ Flk-1+ cells do not show higher expression of myeloid determining genes compared to incommitted HSCs. Quantitative real time PCR analysis of c/EBPα (A) and PU.1 (B) mRNA levels in sorted HSCs and Lin-cKit+Flk-1+ cells. The bars represent the fold change of c/EBPα and PU.1 mRNA levels in Lin-cKit+Flk-1+ cells relative to HSC levels. The results were normalized to HPRT expression and expressed as average fold change±SD (c/EBPα 1.3±0.17 n = 6; PU.1 1.3±0.147 n = 6). (0.26 MB TIF) [file pone.0001338.s002.tif]

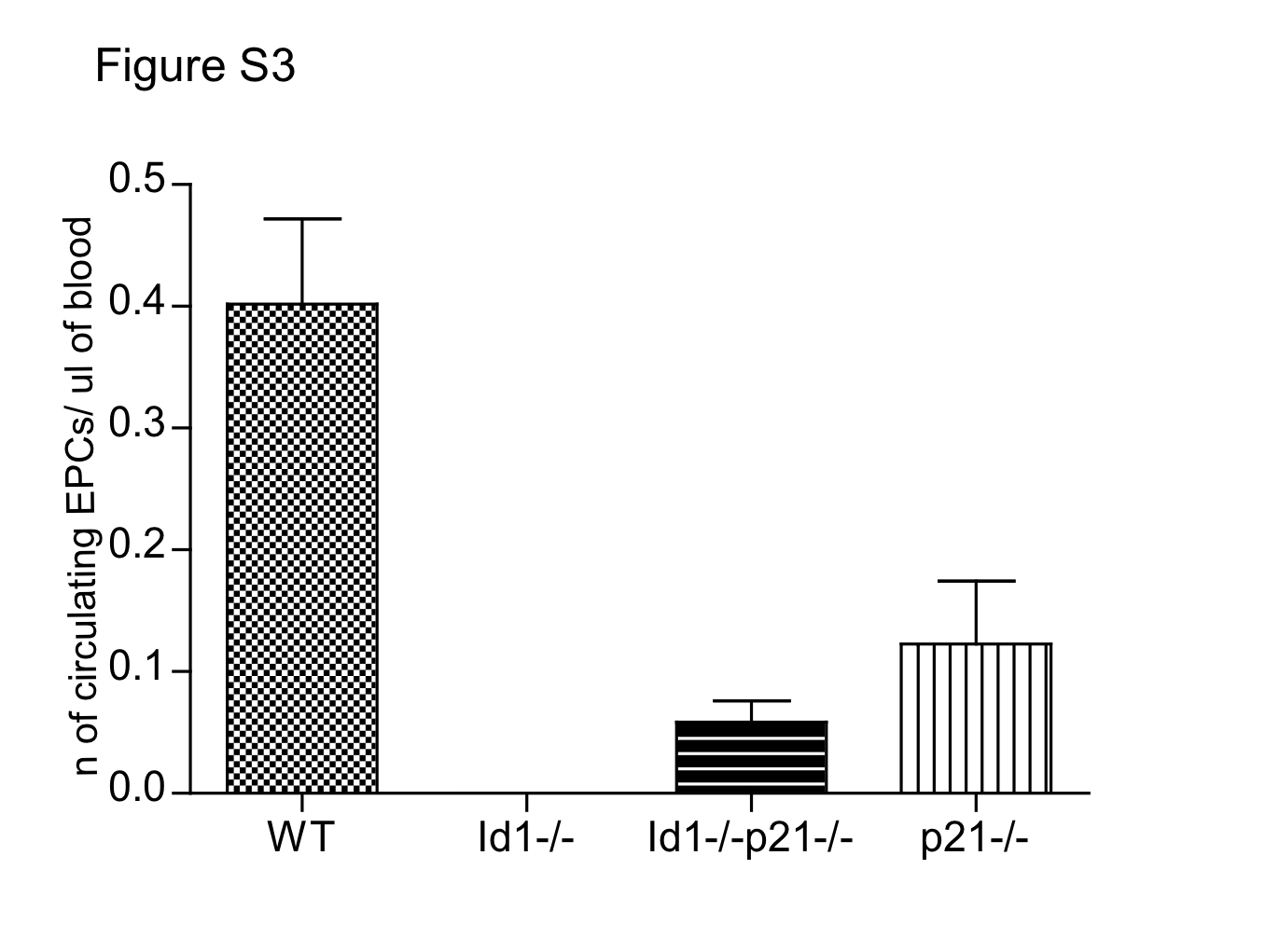

Supplement: Figure S3 — Ablation of p21 rescues the endothelial differentiation defect of Id1-/- HSCs. Id1-/- mice were sublethally irradiated then transplanted with 20 Lin- cKit+ Sca-1+ CD34- Flk-1- HSCs purified from the BM of the indicated group of mice. The histograms represent the flow cytometry analysis of circulating EPCs in the peripheral blood of Id1-/- mice 4 weeks after transplantation. The bars represent the average number or circulating EPCs (±SEM) per µl of blood. WT HSCs: 0.4±0.007 (n = 5); Id1-/- HSCs: 0.0001±0.0003 (n = 3); Id1-/-p21-/- HSCs: 0.06 (0.13 MB TIF) [file pone.0001338.s003.tif]

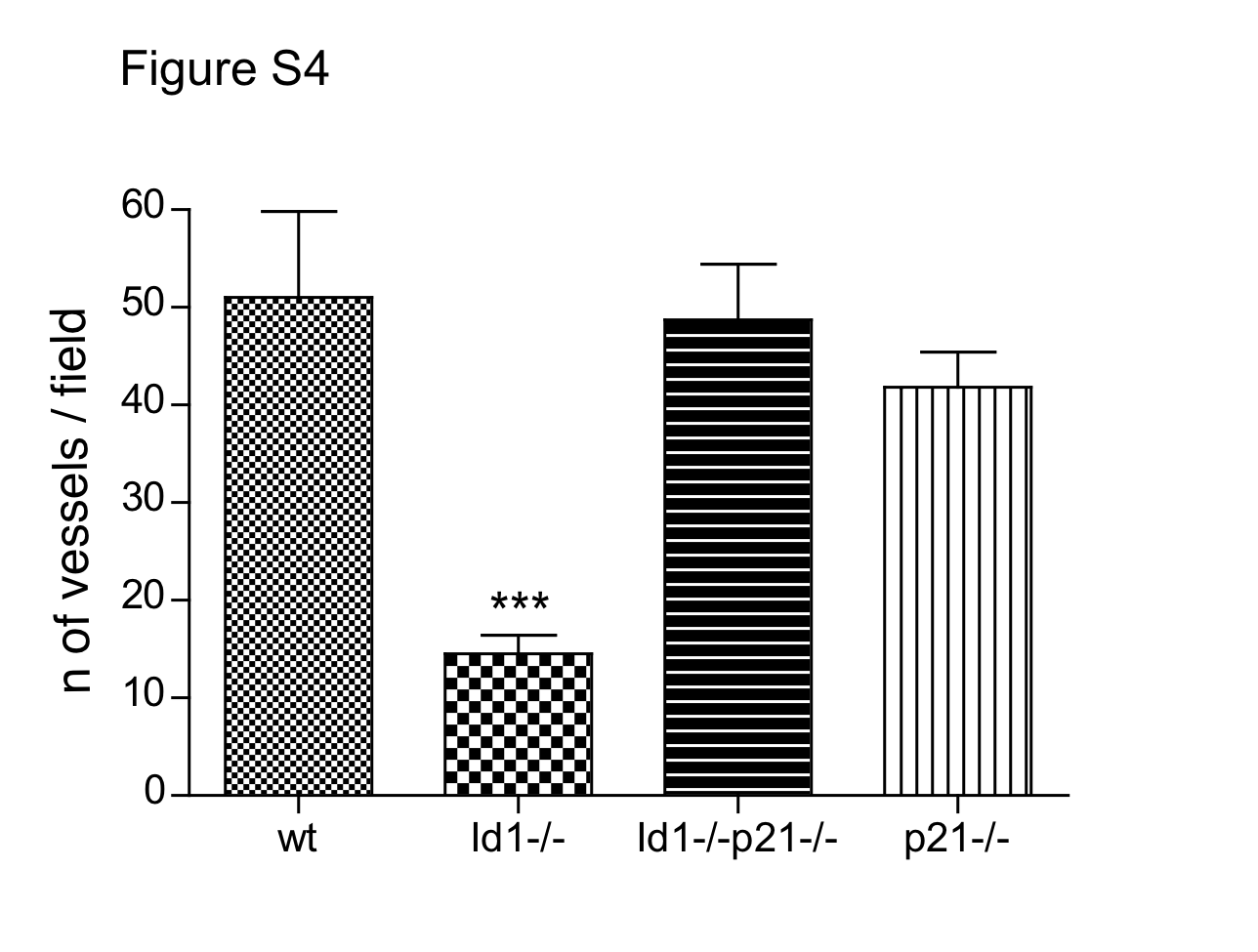

Supplement: Figure S4 — Quantification of vessels in LLC tumors from WT, Id1-/-, Id1-/-p21-/- and p21-/- mice. A minimum 400 vessels were counted from 5 non sequential sections were counted. Average±SEM: WT 51±8.8; Id1-/- 14.56±1.9; Id1-/-p21-/- 48.78±5.7 and p21-/- 41.89±3.55. (0.12 MB TIF) [file pone.0001338.s004.tif]
